# The enhancing effects of selenomethionine on harmine in attenuating pathological cardiac hypertrophy via glycolysis metabolism

**DOI:** 10.1111/jcmm.70124

**Published:** 2024-10-01

**Authors:** Qi Chen, Wen‐Yan Wang, Qing‐Yang Xu, Yan‐Fa Dai, Xing‐Yu Zhu, Zhao‐Yang Chen, Ning Sun, Chung‐Hang Leung, Fei Gao, Ke‐Jia Wu

**Affiliations:** ^1^ Wuxi School of Medicine Jiangnan University Wuxi Jiangsu P. R. China; ^2^ Department of Physiology and Pathophysiology, State Key Laboratory of Medical Neurobiology School of Basic Medical Sciences, Fudan University Shanghai P. R. China; ^3^ Department of Cardiology, Heart Center of Fujian Province Fujian Medical University Union Hospital Fuzhou Fujian P. R. China; ^4^ State Key Laboratory of Quality Research in Chinese Medicine Institute of Chinese Medical Sciences, University of Macau Macao P. R. China; ^5^ Department of cardiology, Beijing An Zhen Hospital Capital Medical University Chaoyang Beijing P. R. China

**Keywords:** cardiac hypertrophy, combination therapy, glycolysis metabolism, harmine, selenomethionine

## Abstract

Pathological cardiac hypertrophy, a common feature in various cardiovascular diseases, can be more effectively managed through combination therapies using natural compounds. Harmine, a β‐carboline alkaloid found in plants, possesses numerous pharmacological functions, including alleviating cardiac hypertrophy. Similarly, Selenomethionine (SE), a primary organic selenium source, has been shown to mitigate cardiac autophagy and alleviate injury. To explores the therapeutic potential of combining Harmine with SE to treat cardiac hypertrophy. The synergistic effects of SE and harmine against cardiac hypertrophy were assessed in vitro with angiotensin II (AngII)‐induced hypertrophy and in vivo using a *Myh6* ^*R404Q*^ mouse model. Co‐administration of SE and harmine significantly reduced hypertrophy‐related markers, outperforming monotherapies. Transcriptomic and metabolic profiling revealed substantial alterations in key metabolic and signalling pathways, particularly those involved in energy metabolism. Notably, the combination therapy led to a marked reduction in the activity of key glycolytic enzymes. Importantly, the addition of the glycolysis inhibitor 2‐deoxy‐D‐glucose (2‐DG) did not further potentiate these effects, suggesting that the antihypertrophic action is predominantly mediated through glycolytic inhibition. These findings highlight the potential of SE and harmine as a promising combination therapy for the treatment of cardiac hypertrophy.

## 1 BACKGROUND

Pathological cardiac hypertrophy is a prevalent phenomenon observed in various cardiovascular diseases such as hypertension, aortic stenosis, and ischemic injury, often leading to heart failure, arrhythmias, and even sudden cardiac death.^1^, ^2^ Current treatment options for cardiac hypertrophy primarily involve the use of β‐blockers, calcium antagonists, and angiotensin‐converting enzyme (ACE) inhibitors via enhancing cardiac systolic and diastolic function. However, these medications can be limited by side effects such as fatigue, cold extremities, and other adverse reactions, necessitating alternative treatment strategies.^3^

Harmine, a β‐carboline alkaloid with diverse pharmacological properties^4^, ^5^ has been demonstrated to inhibit nuclear factor‐kappa B (NF‐κB) activity, potentially alleviating cardiac hypertrophy. However, long‐term treatment and high dosages required for cardiac hypertrophy therapy limit its application.^1^ Selenium, an essential trace element in humans, plays a crucial role in biological processes.^6^, ^7^ Selenium deficiency has been linked to cardiomyopathy,^8^ and dietary supplementation with selenomethionine (SE), a key organic selenium source, has shown promise in enhancing cardiomyocyte function by modulating the AKT signalling pathway.^9^ Activation of the AKT signalling pathway enhances the transcriptional activity of NF‐κB, whereas inhibition of AKT disrupts NF‐κB activity, suggesting that co‐treatment of SE and harmine might exert a synergistic effect against cardiac hypertrophy.^10^, ^11^, ^12^, ^13^ Combination therapy using natural products has been shown to be more effective than single‐agent therapy.^14^, ^15^ In our study, we investigate the combined effects of SE and harmine on cardiac hypertrophy, revealing significant reductions in hypertrophy‐related targets. Our findings, validated in in vitro with angiotensin II (AngII)‐induced hypertrophy and in vivo using a *Myh6* ^*R404Q*^ mouse model, underscore the therapeutic potential of this dual approach, particularly through modulation of glycolytic metabolism. This highlights a novel strategy leveraging natural products to manage cardiac hypertrophy effectively.

## 2 EXPERIMENTAL SECTION

### 2.1 Cell culture and treatment

AC16 human left ventricular cardiomyocytes and human embryonic stem cell‐derived cardiomyocytes (hESC‐CMs) were utilized in this study. AC16 cells were cultured in Dulbecco's modified Eagle's medium (DMEM) supplemented with 10% fetal bovine serum (FBS) and 1% penicillin–streptomycin. Upon reaching suitable confluence, AC16 cells were dissociated using 0.05% EDTA/Trypsin. Cardiac differentiation of hESC‐CMs was carried out following a previous study described.^1^ The detailed information of hESC‐CMs were listed in supplementary materials. All cells were maintained in a 37°C incubator with 5% CO_2_.

The specific administration for each group were as follows:

Control group: Cardiomyocytes treatment with 0.1% DMSO for 24 h; Model group: Cardiomyocytes treated with 1 μM AngII (MCE, Cat#HY‐13948) for 24 h; Treatment group: Cardiomyocytes were pre‐treated with SE and harmine (TargetMol, Cat# T1711 and T1658, respectively), either individually or in combination for 3 h followed by co‐treatment with 1 μM AngII for 24 h. Intervention group: Cardiomyocytes were pre‐treated with SE and harmine (TargetMol, Cat# T1711 and T1658, respectively), together with 2‐Deoxy‐D‐glucose (2‐DG) (TargetMol, Cat# T6742), for 3 h. Subsequently, the cells were co‐treated with 1 μM AngII for 24 h.

### 2.2 Animal model and group

*Myh6* ^*R404Q*^ mice is a good model for studying cardiac hypertrophy, the mice were purchased from GemPharmatech Co., Ltd. Mice were maintained in a controlled 12/12‐h light/dark cycle with unrestricted access to a standard rodent chow diet and water. Harmine and SE from TargetMol (Cat#T1711 and #T1658) were thoroughly integrated into their regular diet at concentrations of 0.05% and 0.25%, respectively. At the age of three weeks, genotyping was performed to distinguish between wild type and *Myh6* ^*R404Q*^ littermates. By six weeks, the mice were randomly divided into five groups: a control group consisting of wild type littermates fed with regular feed (*n* = 6), a model group of *Myh6* ^*R404Q*^ mice fed with regular feed (*n* = 6), a harmine group of *Myh6* ^*R404Q*^ mice fed with a 0.05% harmine diet (n = 6), a SE group of *Myh6* ^*R404Q*^ mice fed with a 0.25% SE diet (n = 6), and a co‐treatment group of *Myh6* ^*R404Q*^ mice fed with both 0.05% harmine and 0.25% SE diet (n = 6). Body weight and food intake were monitored weekly during the treatment period. After four weeks of treatment, the mice were euthanized, and their hearts were promptly excised. The majority of the myocardial samples were fixed in formalin for histological examination, while a small portion of the left ventricular apex was immediately frozen in liquid nitrogen and preserved at −80°C for future analysis. The experimental protocols were fully approved by the Institutional Animal Care and Use Committee (IACUC) of Jiangnan University, under approval No. 20230315 m0401020.

### 2.3 Echocardiography assessment

Transthoracic echocardiography was performed using a Vevo 3100 Imaging System (Visual Sonics) at both the onset and conclusion of the four‐week experiment.^16^ Echocardiographic assessments were made from M‐mode images captured in both the long‐axis and short‐axis views. Key parameters evaluated included ejection fraction (EF), fractional shortening (FS), and left ventricular mass (LV mass). Additionally, measurements were taken for left ventricular anterior wall end‐systolic thickness (LVAW;s), left ventricular anterior wall end‐diastolic thickness (LVAW;d), left ventricular posterior wall end‐systolic thickness (LVPW;s), and left ventricular posterior wall end‐diastolic thickness (LVPW;d), to assess overall left ventricular function and myocardial wall thickness.

### 2.4 Western blot analysis

The extraction of total protein from AC16 cells was carried out according to the previous study.^15^ Subsequently, 30 μg of protein from each sample were loaded onto 10% SDS‐PAGE gels for electrophoresis, followed by transfer onto PVDF membranes (Millipore) at a constant current of 300 mA for 1 h. The membranes were then blocked using 5% skim milk (BD Pharminigen, Cat#232100) in TBST (containing 0.1% Tween 20) at room temperature for 2 h. Overnight incubation with primary antibodies was performed at 4°C. Following primary antibody incubation, the membranes were washed three times with TBST for 10 min each. After washing, the membranes were incubated with HRP‐conjugated Affinipure Goat Anti‐Mouse/Rabbit antibodies (1:10000 dilution, APExIO, Cat#K1221/K1223) at room temperature for 1.5 h. Protein bands were detected using an ECL kit (Vazyme, Cat#E422‐02) and visualized on a Tanon 4600SF imaging system. Quantitative analysis of the immunoreactive bands was achieved using ImageJ software.^15^

### 2.5 Histological analysis

Hearts were excised, thoroughly rinsed, and fixed in 10% formaldehyde overnight. After fixation, the hearts were embedded in paraffin and sectioned at a thickness of 4 μm. The sections underwent sequential staining with haematoxylin and eosin (H&E) (Wanleibio; Cat#WLA51a), Masson's trichrome (Wanleibio; Cat#WLA045a), and wheat germ agglutinin (WGA) (Servicebio, Cat#L4895). Imaging of the tissue slides was performed using a Slide Scanner System (3DHISTECH) at a magnification of 400×. Cardiomyocyte cell size was quantified using ImageJ software.

### 2.6 Immunofluorescence staining

AC16 cells were reseeded onto 15 mm circular coverslips in 12‐well plates using 10% FBS/DMEM medium. Following a 24 h co‐treatment, cardiomyocytes were fixed with 4% paraformaldehyde for 15 min and permeabilized with 0.1% PBST (Triton X‐100) for 10 min. After blocking with goat serum for 30 min, the cardiomyocytes were incubated with primary antibody Anti‐MYH7 (1:400, Biolab, Cat#bs‐24941R) at 4°C overnight. Subsequently, staining with AlexaFluor488 antibodies (1:2000, Yeasen, Cat#33906ES60) was performed at room temperature for 90 min. Finally, DAPI (1:1000, Yeasen, Cat#40728ES03) was added for an additional 20 min to stain the nuclei. The fluorescence image of MYH7 in the cells was acquired using a fluorescent microscope (Echo Revolve).

### 2.7 Cell viability assay

Cardiomyocytes were seeded in 96‐well plates at a density of 5000 cells per well and cultured overnight. Harmine (0 to 300 μM) and SE (0 to 400 μM) were dissolved in DMSO and mixed with the growth medium, with the final DMSO concentration not exceeding 0.1%. Serial dilutions of each compound were added to the respective wells. The microplate was then incubated in a humidified incubator at 37°C and 5% CO_2_ for 24 h. After incubation, cells were treated with 10% CCK‐8 cell counting kit (Vazyme Cat#A311) for 3 h. Subsequently, the plate was scanned, and the absorbance at 450 nm was measured using a microplate reader (BioTek). Curve fitting and IC_50_ calculation were conducted using Prism 8 software (GraphPad).

### 2.8 RNA extraction and quantitative real‐time PCR

Total RNA was extracted from AC16 cells using Trizol (Vazyme), and 1 μg of RNA was reverse‐transcribed into cDNA using the HiScript III All‐in‐one RT SuperMix for qPCR (Vazyme). Quantitative RT‐PCR was performed with specific primers and ChamQ Universal SYBR qPCR Master Mix (Vazyme) on the LightCycler 96 PCR System (Roche). The relative gene expression levels were normalized to 36B4 in cardiomyocytes. The relative mRNA levels were analysed using the 2^−ΔΔCt^ method. Primer sequences used in this study are listed in Table S1.

### 2.9 RNA‐sequencing

The RNA sequencing was carried out according to the previous study.^17^ Total RNA was extracted, and mRNA was subsequently isolated and reverse‐transcribed into cDNA. A stranded RNA sequencing library was prepared as described previously. The mRNA was enriched and fragmented into short fragments. A random primer was employed to synthesize a single strand of cDNA using the mRNA fragments as templates. After synthesizing and purifying double‐stranded cDNA, the sample purification efficiency was assessed. Subsequently, sequence analysis was performed using an Illumina NovaSeqTM 6000 (Illumina). Following the generation of the final transcriptome, differential gene expression identification and estimation of expression abundance were conducted using OmicsBean. Genes with a log2 (fold change) >2 and a false discovery rate (FDR) <0.05 were considered significantly differentially expressed between the two conditions.

### 2.10 Untargeted metabolomic analysis

The ultrahigh performance liquid chromatography‐tendem mass spectrometry (UHPLC–MS/MS) analysis was carried out according to the previous study.^17^ Cardiomyocytes were mixed with an 80% methanol aqueous solution, quick‐frozen in liquid nitrogen, and sonicated on ice. The mixture was then centrifuged at 5000 rpm at 4°C for 1 min. The supernatant was collected and lyophilized into a dry powder. Subsequently, 10% methanol solution was added to dissolve the sample for liquid chromatography‐mass spectrometry (LC–MS) analysis (Biozeron, China). Statistical analysis was performed on log2‐transformed metabolite values. Partial least squares discriminant analysis (PLS‐DA analysis) was employed to classify the research objects. To identify significant metabolites, the fold change and variable importance in projection (VIP) values from the PLS‐DA model were utilized. KEGG enrichment analysis was employed to further identify pathways that exhibited a high correlation with metabolite differences.

### 2.11 Measurements of glucose 6 phosphate levels

Cellular glucose 6 phosphate (G6P) levels were measured using a G6P Assay Kit with WST‐8 (Beyotime, #S0185) according to the manufacture's instruction. The absorbance at 450 nm was measured using a multimode plate reader (PerkinElmer). The samples were homogenized using Rapid Gold BCA Protein Assay Kit (ThermoFisher, #A53226).

### 2.12 Measurements of ATP levels

Cellular ATP levels were determined using an Enhanced ATP Assay Kit (Beyotime, #S0027) according to the manufacture's instruction. Luminescence was detected using a multimode plate reader. The samples were homogenized using Rapid Gold BCA Protein Assay Kit.

### 2.13 Measurement of NAD ^+^ and NADH levels

Cellular nicotinamide adenine dinucleotide (NAD^+^) and nicotinamide adenine dinucleotide + hydrogen (NADH) levels were determined using a NAD^+^/NADH Assay Kit with WST‐8 (Beyotime, #S0175) according to the manufacture's instruction. The absorbance at 450 nm was measured using a multimode plate reader. The ratio of NAD^+^/NADH was calculated by measuring the total NAD content and the content of NAD^+^ after subtracting NADH. The samples were homogenized using Rapid Gold BCA Protein Assay Kit.

### 2.14 Measurement of glyceraldehyde‐3‐phosphate dehydrogenase activity

Cellular glyceraldehyde‐3‐phosphate dehydrogenase (GAPDH) activity was measured using a GAPDH Activity Assay Kit (Solarbio, #BC2215). The supernatant was collected and measured according to the manufacturer's instruction. The absorbance at 340 nm was measured using a multimode plate reader. The samples were homogenized by liquid volume.

### 2.15 Measurement of Pyruvate content

Cellular pyruvate content was determined using a Pyruvate Assay Kit (Solarbio, #BC2205). The supernatant was collected and measured according to the manufacturer's instruction. The absorbance at 520 nm was measured using a multimode plate reader. The samples were homogenized using Rapid Gold BCA Protein Assay Kit.

### 2.16 Measurement of lactic acid content

Cellular lactic acid (LA) content was determined using a Lactic Acid Content Assay Kit (Boxbio, #AKAC001). The supernatant was collected and measured according to the manufacturer's instruction. The absorbance at 570 nm was measured using a multimode plate reader. The samples were homogenized by liquid volume.

### 2.17 Statistical analysis

Statistical analysis was performed by GraphPad Prism 8. Data were presented as mean ± SEM. Statistical analysis for comparison were evaluated by unpaired two tailed Student's t‐test between two groups or one‐way ANOVA among multiple groups. *p*‐values <0.05 were considered statistically significant.

## 3 RESULTS

### 3.1 Combination synergy assessment of SE and harmine against cardiac hypertrophy

**FIGURE 1:**
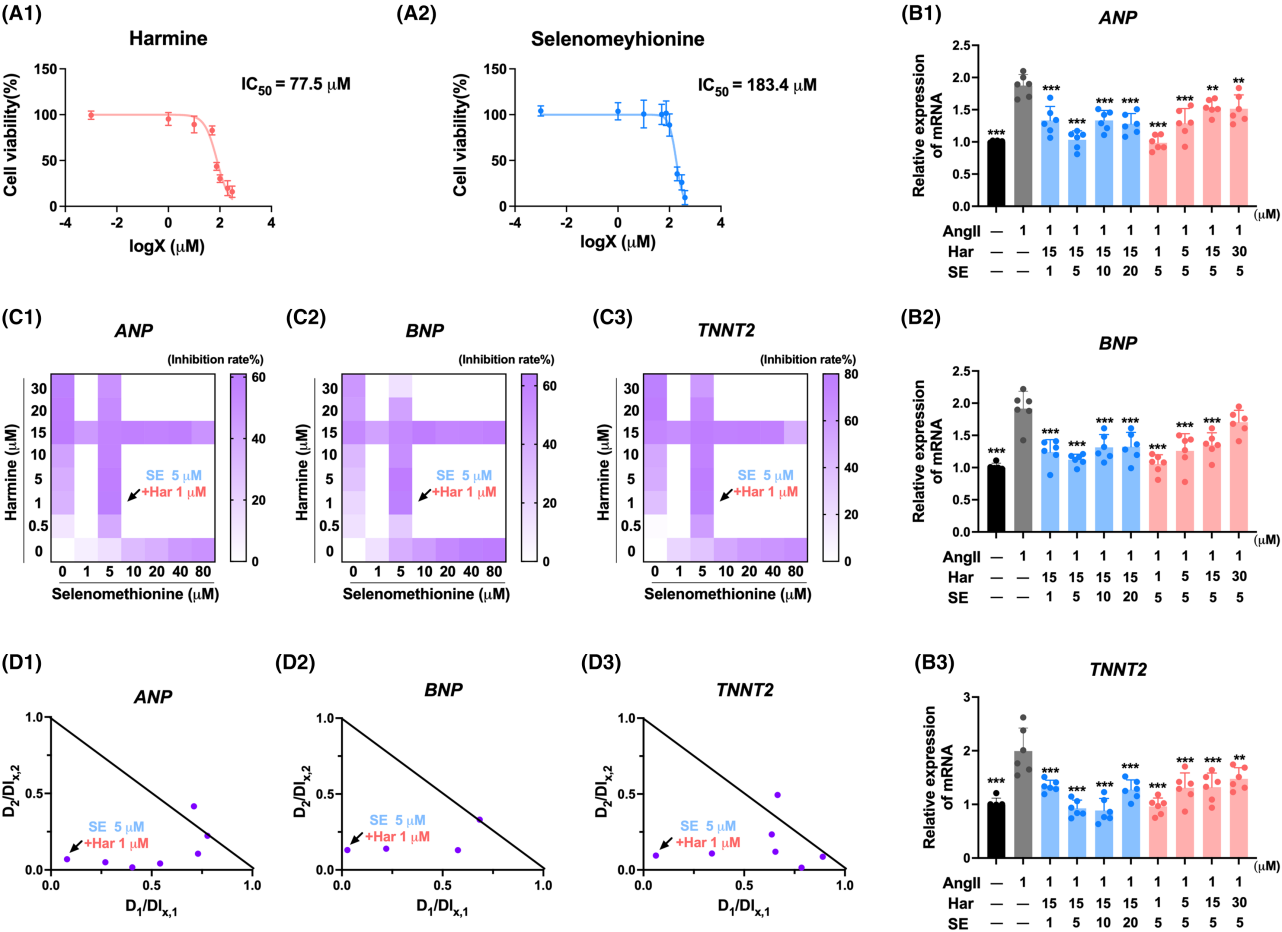
Combination synergy assessment of SE and Harmine against cardiac hypertrophy. (A) Cell viability was measured after treatment of Harmine or SE under various concentrations for 24 h. *n* = 5. (B) qPCR analysis of hypertrophic‐related genes ANP, BNP and TNNT2 in cardiomyocytes (normalized to 36B4 expression). *n* = 6. (C) Dose–response matrix of drug combination measurements representing the inhibition against biomarker genes. (D) Synergistic analysis of the combinatorial landscape based on the Loewe model. Har is an abbreviation of Harmine, SE is an abbreviation of selenomethionine. D1 and D2 refer to the doses of SE and Harmine, respectively. DIx,1 and DIx,2 represent the doses of Harmine and SE, respectively, that would be needed to achieve the same effect as observed in the combination, but when each drug is used alone. Data are presented as mean ± SEM, *p < 0.05, **p < 0.01, ***p < 0.001 versus AngII group by one‐way ANOVA.

To assess the cytotoxicity of SE and harmine, a CCK‐8 assay was performed. The results indicated that treatment with SE (0 to 100 μM) and harmine (0 to 50 μM) for 24 h did not result in significant cytotoxicity in AC16 cells (Figure 1A). These findings suggest that SE and harmine exhibit favourable biocompatibility. Atrial Natriuretic Peptide (ANP), B‐type Natriuretic Peptide (BNP), and Cardiac Troponin T (TNNT2) are biomarkers that are known to increase in pathological cardiac hypertrophy.^18^ To investigate the combined effects of SE and harmine on AngII‐induced hypertrophic cardiomyocytes, the mRNA expression levels of ANP, BNP, and TNNT2 were evaluated after co‐treatment with different concentrations of SE and harmine. The results demonstrated that the co‐administration of SE and harmine significantly attenuated AngII‐induced hypertrophic cardiomyocytes (Figure 1B). Subsequently, a dose–response experiment was conducted to assess whether the combination treatment exhibited greater efficacy compared to the individual agents. Heatmap analysis revealed that harmine effectively restored ANP mRNA levels at 1 and 5 μM when combined with SE at 5 μM. Furthermore, SE (5 μM) exhibited a similar restorative effect on BNP when co‐treated with harmine at 1 or 5 μM. Additionally, the combination of harmine at a concentration of 15 μM with SE at concentrations of 10, 20, and 40 μM, as well as harmine at 1 μM with SE at 5 μM, exhibited a comparable synergistic enhancement of TNNT2 mRNA levels (Figure 1C). The Loewe model is a well‐established method used to evaluate drug interactions by comparing the combined effect of two drugs to the effect expected if each drug were administered individually at doses producing the same outcome. This model is instrumental in determining whether a drug combination exhibits synergistic, additive, or antagonistic interactions.^19^ To further evaluate the synergistic effect against cardiac hypertrophy, the Loewe model was employed. The results revealed that the combined treatment of SE (5 μM) and harmine (1 μM) significantly alleviated AngII‐induced hypertrophic cardiomyocytes and exhibited a promising synergistic effect against cardiac hypertrophy (Figure 1D).

### 3.2 Co‐treatment of SE and harmine reverses AngII‐induced cardiac hypertrophy

**FIGURE 2:**
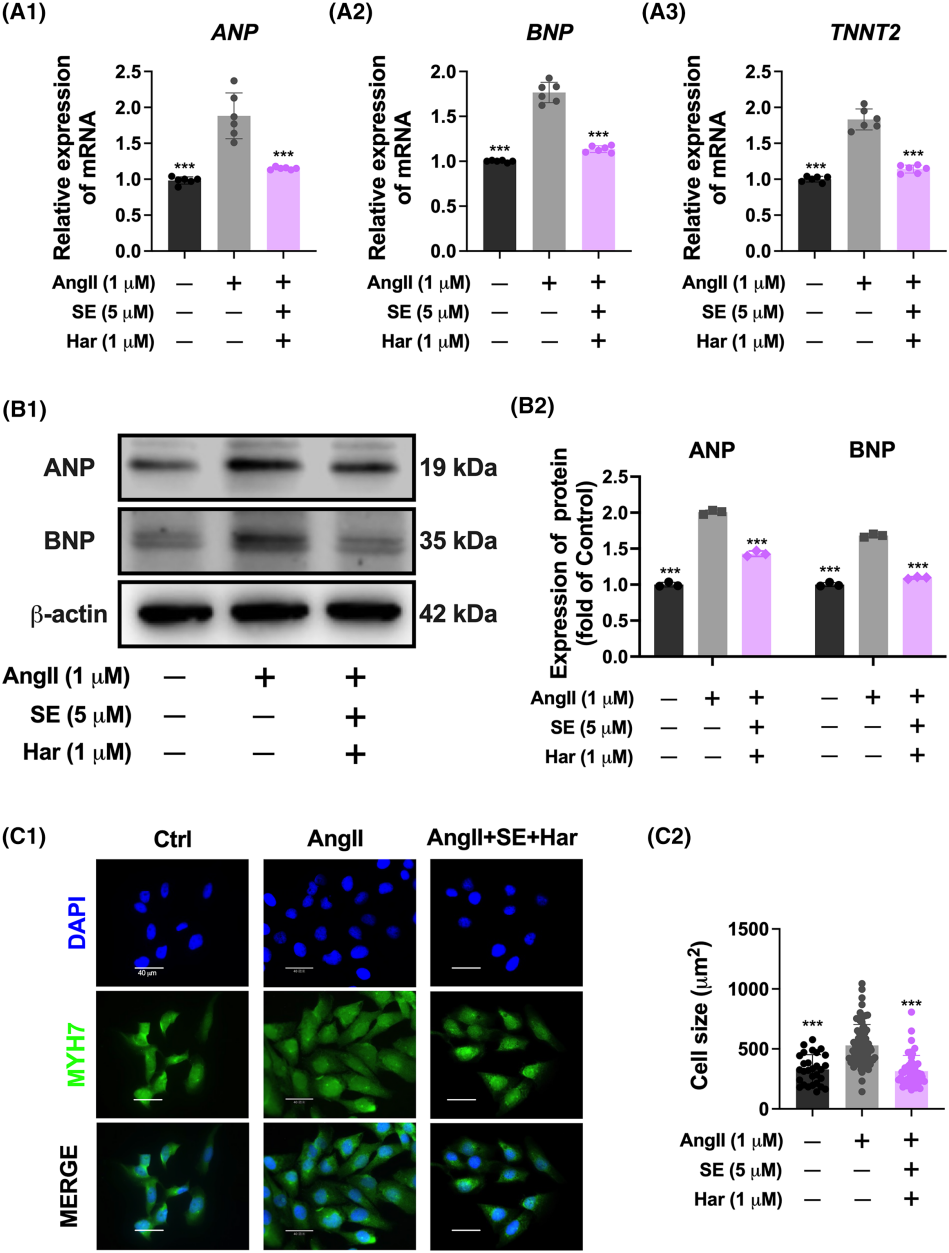
Suppression of AngII‐induced hypertrophic responses by co‐treatment with SE and Harmine. (A) qPCR analysis showing the expression levels of hypertrophic markers ANP, BNP, and TNNT2 in cardiomyocytes, normalized to 36B4 expression. *n* = 6. (B) Western blot analysis demonstrating the protein expression levels of hypertrophic markers ANP and BNP in cardiomyocytes, normalized to β‐Actin expression. *n* = 3. (C) Representative immunofluorescence images of MYH7‐stained for sarcomere marker (green) and DAPI (blue) for nuclei of AC16. Scale bar = 40 μm. Data are presented as mean ± SEM. *p < 0.05, **p < 0.01, ***p < 0.001 versus AngII group by one‐way ANOVA.

Combined treatment of SE (5 μM) and harmine (1 μM) demonstrated a synergistic effect in counteracting cardiac hypertrophy. To further substantiate this conclusion, we assessed hypertrophy markers at both mRNA and protein levels. The results revealed that the co‐treatment successfully reversed the upregulation of ANP, BNP, and TNNT2 mRNA levels in AngII‐induced hypertrophic cardiomyocytes (Figure 2A). A similar reversal was observed at the protein level (Figure 2B). Immunostaining for the cardiomyocyte‐specific marker MYH7 showed that, compared to the control group, the cardiomyocyte area significantly expanded in response to AngII. In contrast, the co‐treated group showed a substantial reduction in cardiomyocyte area, indicating an effective anti‐hypertrophic response (Figure 2C). Further analysis was conducted on the phenotypic and functional impacts of the SE and harmine co‐treatment on AngII‐induced hESC‐CMs. Immunostaining results revealed a significant expansion in the cardiomyocyte area in the AngII‐treated group, which was markedly reduced in the co‐treated group, underscoring the treatment's anti‐hypertrophic efficacy (Figure S1A,B). Cardiac hypertrophy is frequently linked with altered calcium signalling and increased myofilament Ca^2+^ sensitization.^20^ To assess these aspects, calcium transients, which are indicative of cardiomyocyte mechanical contraction, were analysed. The co‐treatment extended the duration of the calcium transient, including peak and decay times, and notably decreased the transient amplitude and frequency in ventricular myocytes, compared to those treated with AngII alone (Figure S1C,D). In summary, co‐treatment of SE (5 μM) and harmine (1 μM) antagonized AngII‐induced cardiomyocyte hypertrophy. These comprehensive findings support that SE and harmine co‐treatment effectively antagonizes Ang II‐induced cardiomyocyte hypertrophy.

### 3.3 Co‐treatment of SE and harmine alleviates cardiac hypertrophy in *Myh6* ^*R404Q*^ mice

**FIGURE 3:**
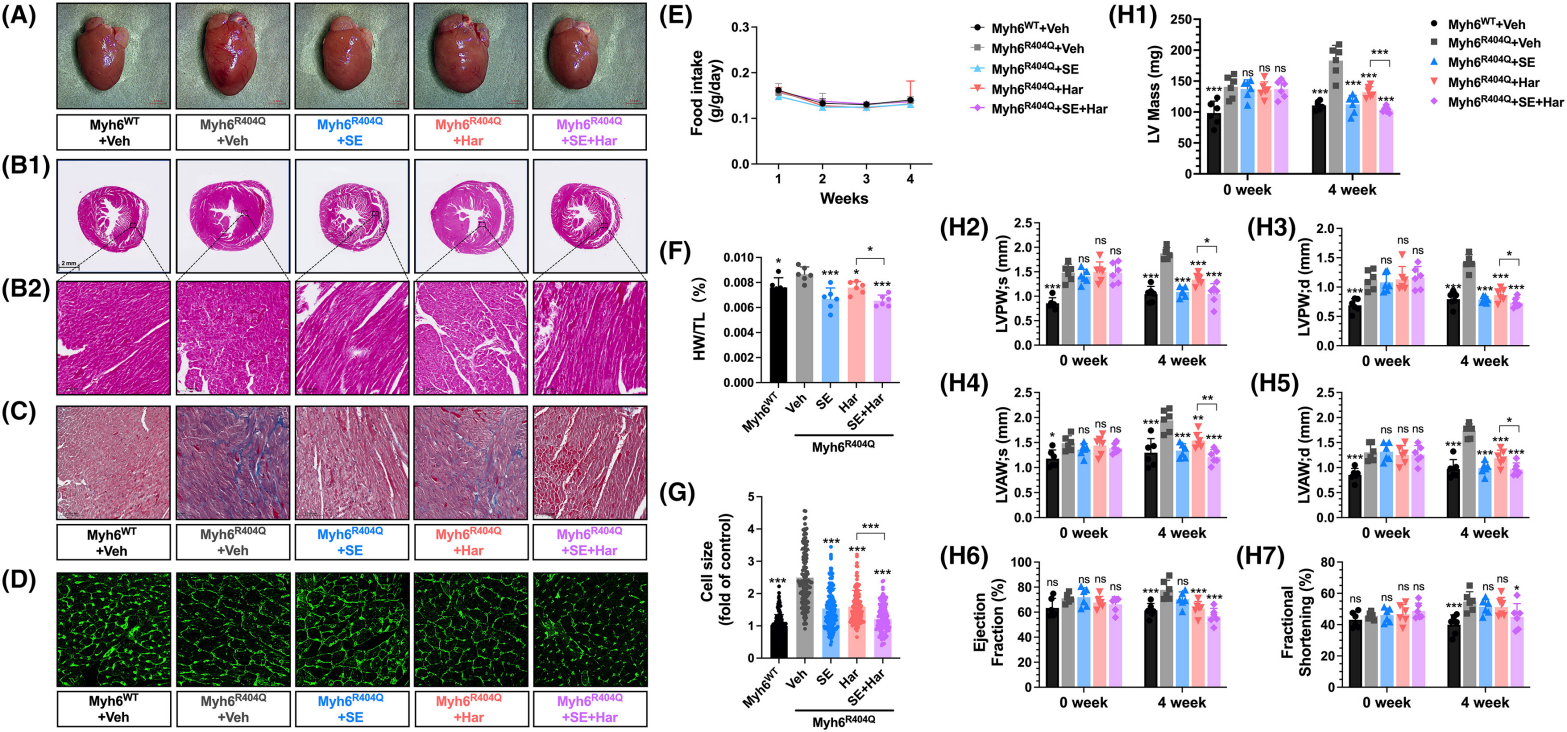
Co‐treatment of SE and Harmine alleviates cardiac hypertrophy in Myh6^R404Q^ mice. (A) Macroscopic view of heart size. (B) Representative images of H&E staining of heart tissues (upper, scale bar: 2 mm), along with high‐magnification views (bottom, scale bar: 100 μm). (C) Masson's trichrome staining of cardiac tissue sections (scale bar: 100 μm). (D) WGA staining of cardiac tissue sections. (E) Weekly food intake of each group during the 4‐week treatment. (F) Quantification of heart weight to body weight ratio in each group. (G) Quantification of WGA staining images. Quantitative data are presented as the mean ± SEM. (H1―7) Measurements of left ventricular anterior wall end systolic thicknesses (LVAW;s), left ventricular anterior wall end diastolic thicknesses (LVAW;d), left ventricular posterior wall end systolic thicknesses (LVPW;s), and left ventricular posterior wall end diastolic thicknesses (LVPW;d), left ventricular mass (LV mass), ejection fraction (EF), and fractional shortening (FS). *n* = 6. Data are presented as mean ± SEM. *p < 0.05, **p < 0.01, ***p < 0.001 versus Myh6^R404Q^ + Veh group by one‐way ANOVA.

To further evaluate the therapeutic efficacy of SE and harmine co‐treatment against cardiac hypertrophy, preliminary echocardiographic assessments were performed on six‐week‐old *Myh6* ^*R404Q*^ mice. At this age, the mice exhibited significantly enlarged hearts. Following four weeks of co‐treatment with SE and harmine, there was a notable reduction in heart size (Figure 3A; Figure S2). Histological assessments using haematoxylin and eosin (H&E) and Masson's trichrome staining revealed that SE and harmine co‐treatment effectively restored the disrupted cardiomyocyte arrangement and mitigated the increase in myocardial tissue fibrosis observed in *Myh6* ^*R404Q*^ mice (Figure 3B,C). Wheat germ agglutinin (WGA) staining indicated an increased cardiomyocyte size relative to the wild‐type (WT) group. However, co‐treatment significantly reversed these pathological changes (Figure 3D,G). No significant differences in food intake were detected between the groups (Figure 3E). Furthermore, the heart weight to tibia length (HW/TL) ratio, which was significantly elevated in the *Myh6* ^*R404Q*^ mice, showed substantial reduction following the SE and harmine intervention (Figure 3F). Echocardiographic evaluations conducted prior to the intervention revealed significant cardiac abnormalities, including increased thickness of both the systolic and diastolic ventricular walls, elevated LV mass, and enhanced EF and FS. These abnormalities deteriorated further over the subsequent four weeks (Figure 3H). Remarkably, the co‐treatment significantly reversed the ventricular wall thickening and reduced LV mass in *Myh6* ^*R404Q*^ mice, while maintaining EF and FS within normal ranges (Figure 3H). Most importantly, this co‐treatment was more effective in reversing the pathological alterations in cardiac structure and function than SE or harmine treatment alone, suggesting its enhanced therapeutic potential in the management of cardiomyopathy in *Myh6* ^*R404Q*^ mice.

### 3.4 Omic analyses in cardiomyocytes after SE and harmine co‐treatment

**FIGURE 4:**
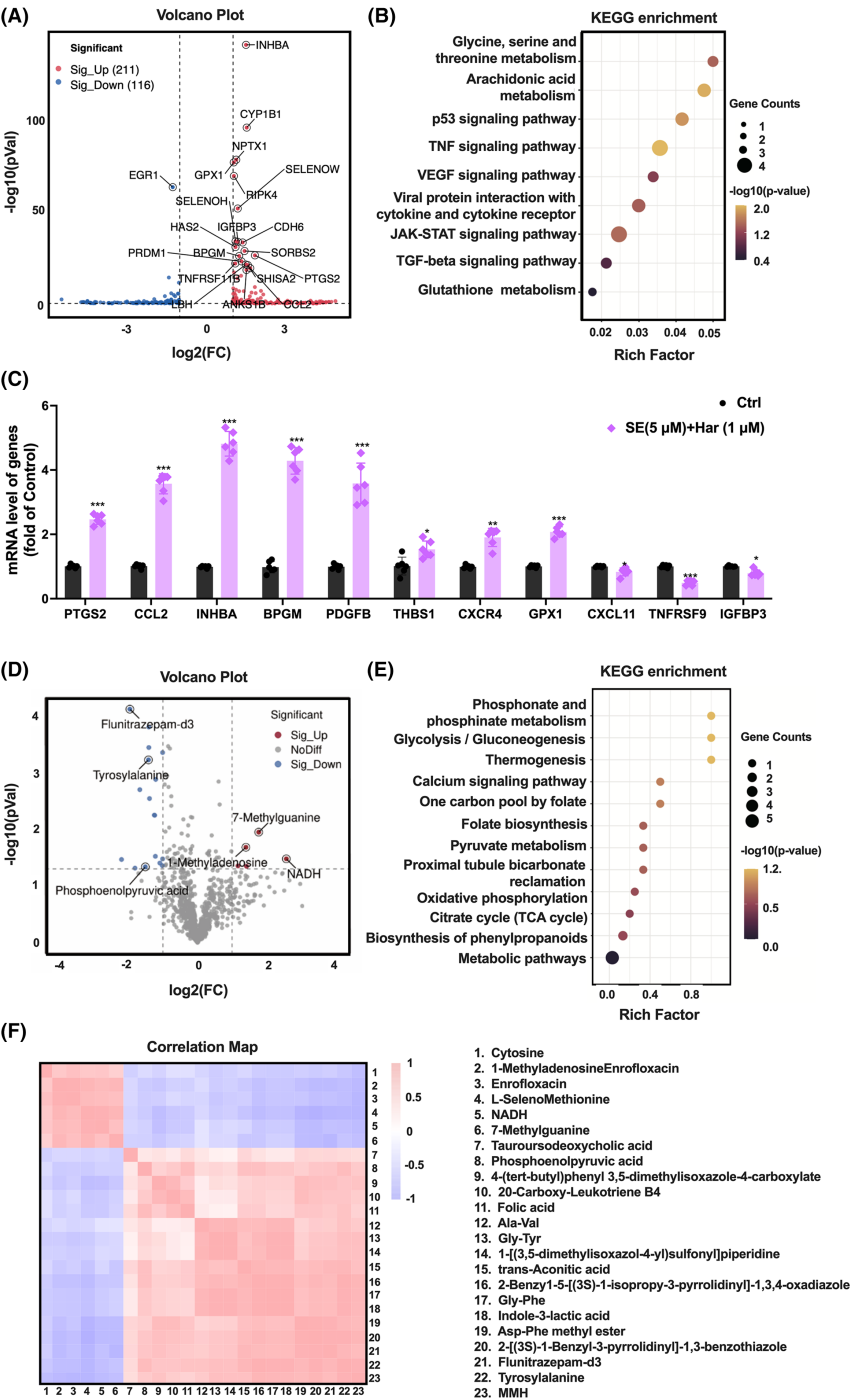
Omic analysis of AngII‐induced hypertrophic after co‐treatment. (A) Volcano plot of the transcriptome showing the significantly up‐regulated and down‐regulated genes. (B) KEGG pathway enrichment analysis of differentially expressed transcripts. (C) Quantitative real‐time PCR analysis of the mRNA expression of PTGS2, CCL2, INHBA, BPGM, PDGFB, THBS1, CXCR4, GPX1, CXCL11, TNFRSF9, IGFBP3 in AC16 cells at 24 h after the co‐treatment. Normalized to 36B4 expression. *n* = 6. Data are presented as mean ± SEM. *p < 0.05, **p < 0.01, ***p < 0.001 versus control group by two‐tailed Student's test. (D) Volcano plot for changed metabolite in cardiomyocytes at 24 h after co‐treatment. Red represented the upregulated metabolites and blue represented the downregulated metabolites. (E) KEGG pathway enrichment analysis of differentially changed metabolites. (F) Clustering heat map for 22 differential metabolites.

To investigate the underlying mechanism of SE and harmine co‐treatment in preventing cardiomyocyte hypertrophy, we conducted RNA‐sequencing (RNA‐Seq) to analyse genome changes in the AC16 cells. The volcano plot demonstrated that 327 transcripts exhibited significant differential expression (DEG) with a fold change of 2 (*p* < 0.05). Among them, 211 transcripts were significantly down‐regulated, while 116 transcripts were significantly up‐regulated in the co‐treatment group compared to the control group (Figure 4A). Through KEGG pathway enrichment analysis of the DEGs, we identified several keys signalling pathways affected by co‐treatment of SE and harmine in cardiomyocytes. These pathways include glycine, serine, and threonine metabolism; arachidonic acid metabolism; the p53 signalling pathway; the tumour necrosis factor (TNF) signalling pathway; the vascular endothelial growth factor (VEGF) signalling pathway; viral protein interaction with cytokines and cytokine receptors; the Janus kinases Signal Transducers and Activators of Transcription (JAK–STAT) signalling pathway; the TGF‐β signalling pathway; and glutathione metabolism (Figure 4B). To further support these findings, we performed quantitative RT‐PCR to validate the RNA‐seq results on critical genes (Figure 4C). The results demonstrated that co‐treatment up‐regulated the expression of PTGS2, CCL2, INHBA, BPGM, PDGFB, THBS1, CXCR4, and GPX1. Conversely, the expression of IGFBP3, CXCL11, and TNFRSF9 was significantly down‐regulated, consistent with the RNA‐seq results. Based on the enriched transcriptomic pathways observed in our transcriptome data, we performed metabolic studies to identify key metabolites involved in cardiomyocytes after co‐treatment of SE and harmine. We conducted untargeted metabolomic analysis to assess the distinct metabolic profiles in AC16 cells after co‐treatment of SE and harmine for 24 h. The volcano plot displayed 17 down‐regulated metabolites and 5 up‐regulated metabolites between the control and co‐treatment groups (Figure 4D). Interestingly, we observed a significant decrease in phosphoenolpyruvic acid and an up‐regulation of NADH, both of which are closely associated with energy metabolism. To gain further insights, we conducted KEGG pathway enrichment analysis of the significantly altered metabolites (*p* < 0.05 and fold change ≥2) (Figure 4E). The correlation heatmap analysis revealed the relationships among 22 significantly altered metabolites in the co‐treatment group compared to the control group (Figure 4F). The analysis highlighted phosphonate and phosphinate metabolism, as well as glycolysis/gluconeogenesis as the primarily enriched pathways after co‐treatment of SE and harmine in cardiomyocytes.

### 3.5 Co‐treatment alleviated cardiac hypertrophy mainly through inhibiting glycolysis pathway

**FIGURE 5:**
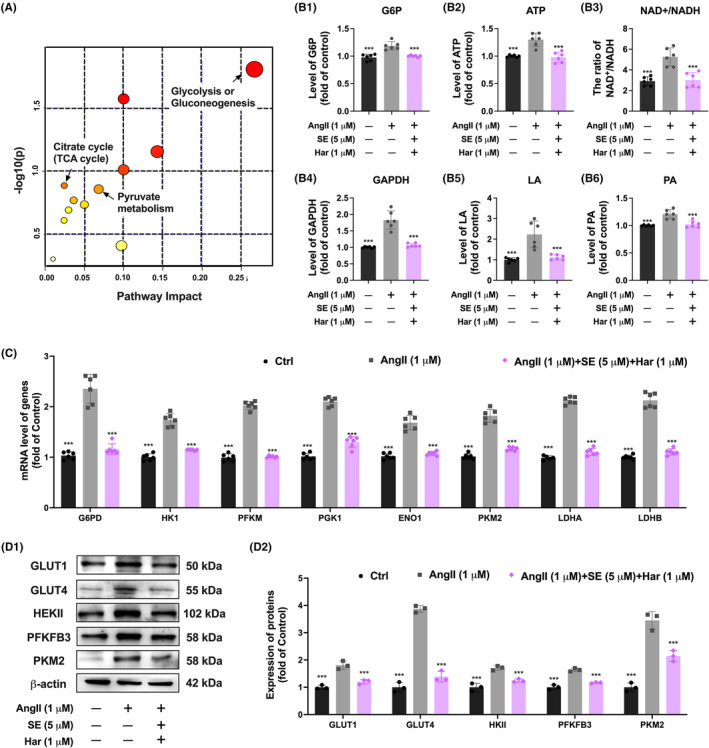
Co‐treatment of SE and Harmine inhibited glycolysis metabolism in AngII‐induced hypertrophic cells. (A) Joint pathway analysis of metabolites and transcripts that significantly changed in AC16 by MetaboAnalyst. (B) Quantitative analysis of G6P, ATP, NAD^+^/NADH ratio, GAPDH activity, pyruvate (PA) and lactic acid (LA). *n* = 6. (C) Quantitative real‐time PCR analysis of the mRNA expression of glycolysis targeted genes G6PD, HK1, PFKM, PGK1, ENO1, PKM2, LDHA and LDHB after co‐treatment of SE and Harmine. Normalized to 36B4 expression, *n* = 6. (D) Western blot analysis demonstrating the protein expression levels of glycolysis pathway markers GLUT1, GLUT4, HEKII, PFKFB3 and PKM2 in cardiomyocytes, normalized to β‐Actin expression. *n* = 3. Data are presented as mean ± SEM. *p < 0.05, **p < 0.01, ***p < 0.001 versus AngII group by one‐way ANOVA.

To investigate the correlation between different metabolites and DEGs after co‐treatment of SE and harmine in AngII‐induced hypertrophic cardiomyocytes, we conducted integrated pathway analysis by combining metabolomics and gene expression data. The analysis revealed that glycolysis/gluconeogenesis was the primarily enriched metabolic pathway (Figure 5A). We demonstrated that co‐treatment with SE and harmine significantly decreased key metabolic markers, including LA, PA, G6P, the NAD^+^/NADH ratio, the activity of GAPDH, and ATP production in AngII‐induced cardiac hypertrophy models (Figure 5B). Mechanistic investigations revealed significant reductions in the expression levels of several critical enzymes within the glycolysis pathway, including G6PD (glucose‐6‐phosphate dehydrogenase), HK1 (hexokinase 1), PFKM (phosphofructokinase, muscle), PGK1 (phosphoglycerate kinase 1), ENO1 (enolase 1), PKM2 (pyruvate kinase M2), LDHA (lactate dehydrogenase A), and LDHB (lactate dehydrogenase B), in cardiac hypertrophic cells treated with SE and harmine (Figure 5C). At the protein level, a corresponding restoration of enzymes including GLUT1, GLUT4, HEKII, PFKFB3 and PKM2 was observed, further underscoring the regulatory impact of the co‐treatment on glycolytic activity (Figure 5D). These findings emphasize the potential of targeting metabolic pathways to regulate cardiac hypertrophy, proposing a novel therapeutic direction.

**FIGURE 6:**
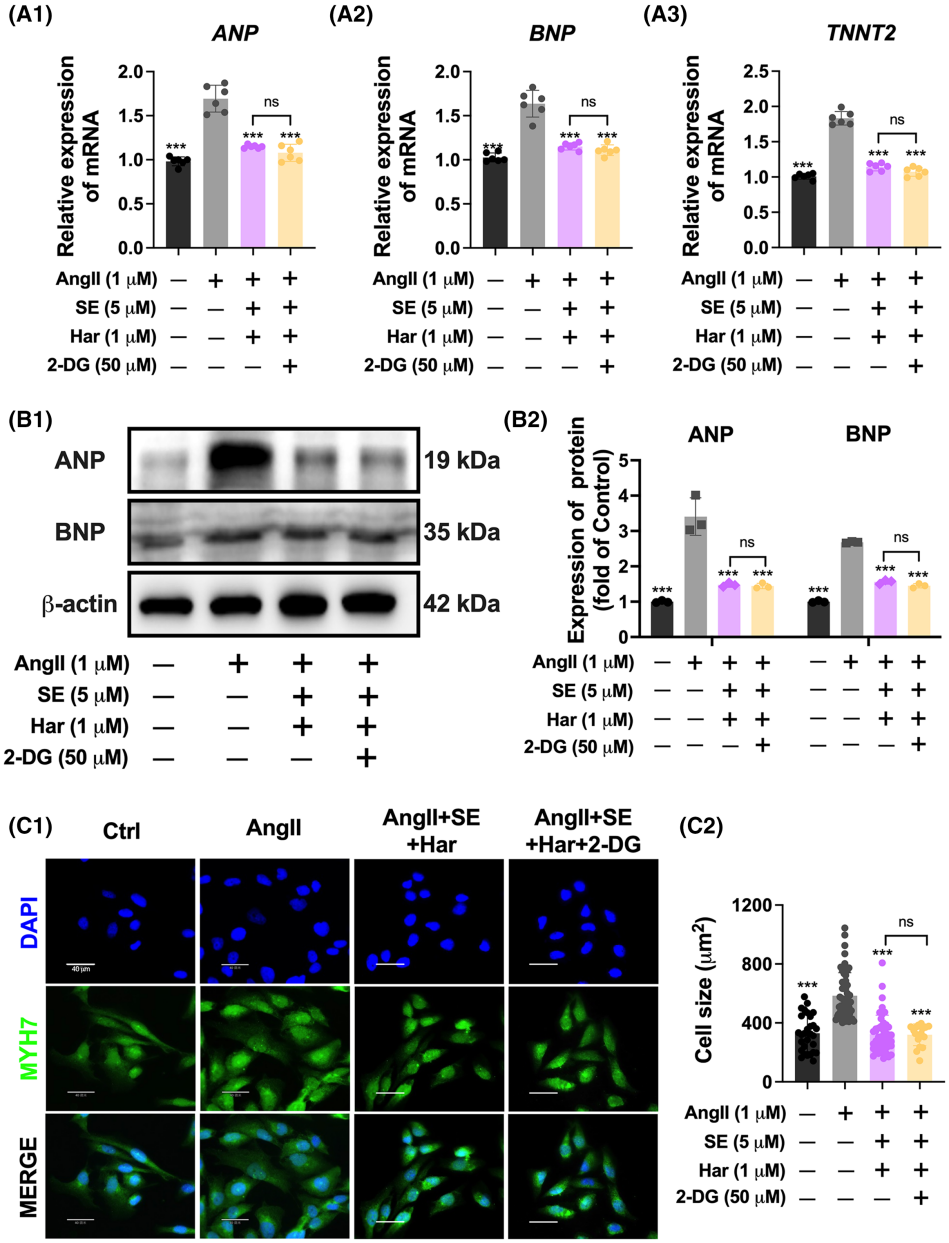
Co‐treatment of SE and Harmine attenuates AngII‐induced hypertrophic via glycolysis pathway. (A) Quantitative real‐time PCR analysis showing the expression levels of hypertrophic markers ANP, BNP, and TNNT2 in cardiomyocytes, normalized to 36B4 expression. *n* = 6. (B) Western blot analysis demonstrating the protein expression levels of hypertrophic markers ANP and BNP in cardiomyocytes, normalized to β‐Actin expression. *n* = 3. (C) Representative immunofluorescence images of MYH7‐stained for sarcomere marker (green) and DAPI (blue) for nuclei of AC16. Scale bar = 40 μm. Data are presented as mean ± SEM. *p < 0.05, **p < 0.01, ***p < 0.001 versus AngII group by one‐way ANOVA.

**FIGURE 7:**
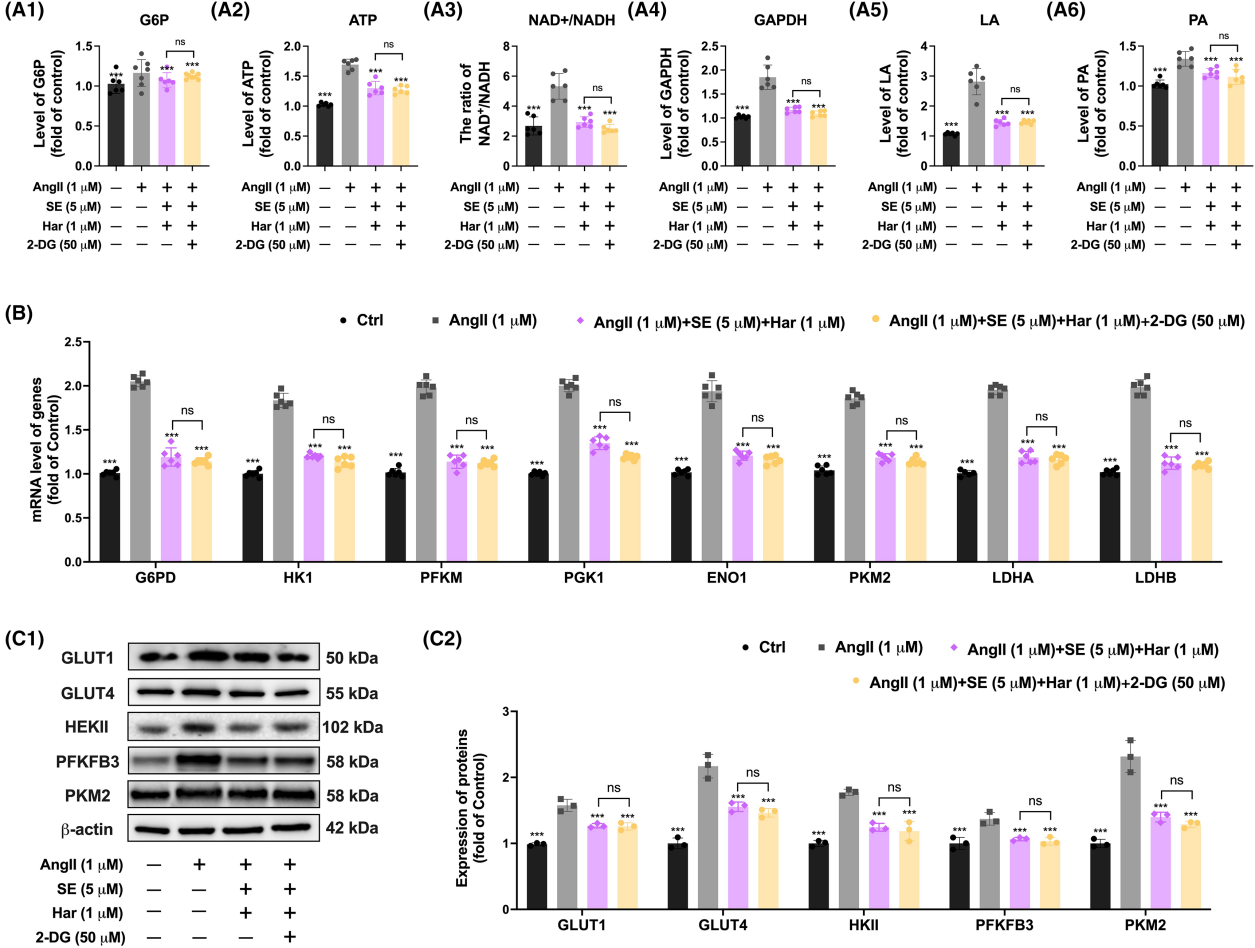
Glycolysis inhibition as atherapeutic strategy for AngII‐induced cardiac hypertrophy. (A) Quantitativeanalysis of G6P, ATP, NAD^+^/NADH ratio, GAPDH activity, PA and LA. *n* = 6. (B) Quantitative real‐time PCR analysis of the mRNA expression ofglycolysis targeted genes G6PD, HK1, PFKM, PGK1, ENO1, PKM2, LDHA and LDHBafter co‐treatment of SE and Harmine in AngII induced AC16 cells for 24 h. Normalized to 36B4 expression, *n* = 6. (C) Western blot analysis demonstrating the protein expression levels ofglycolysis pathway markers GLUT1, GLUT4, HEKII, PFKFB3, and PKM2 incardiomyocytes, normalized to β‐actin expression. *n* = 3. Data are presented asmean±SEM. *p < 0.05, **p < 0.01,***p < 0.001 versus AngII group by one‐way ANOVA.

2‐DG acts as a glycolysis inhibitor by competitively inhibiting the conversion of glucose to glucose‐6‐phosphate at the phosphoglucoisomerase level.^21^ To examine the impact of SE and harmine co‐treatment on the glycolysis pathway, 2‐DG was employed to inhibit this metabolic route. Importantly, the changes in biomarkers of cardiac hypertrophy induced by the co‐treatment of SE and harmine were found to be comparable to those induced by co‐treatment of SE, harmine and 2‐DG in cardiac hypertrophic cells (Figure 6A,B). Furthermore, a consistent reduction in cardiomyocyte area under these conditions was observed (Figure 6C). These observations suggest that the combined therapeutic intervention of SE and harmine can significantly suppress cardiac hypertrophy, with the anti‐hypertrophic effects likely through the modulation of glycolysis metabolism. To further validate the crucial role of glycolysis in the treated cardiomyocytes, significant reductions were observed in key metabolic markers such as LA, PA, G6P, the NAD^+^/NADH ratio, the activity of GAPDH, and ATP production in AngII‐induced cardiac hypertrophy. Additionally, when cardiac hypertrophic cells co‐treatment with 2‐DG, the inhibition glycolysis metabolism caused by SE and harmine was not enhanced by 2‐DG (Figure 7A). Similarly, the expressions of several critical enzymes within the glycolysis pathway, including G6PD, HK1, PFKM, PGK1, ENO1, PKM2, LDHA, and LDHB, which were altered by the co‐treatment, were not further inhibited by 2‐DG (Figure 7B). At the protein level, a similar reversal was observed under the same conditions (Figure 7C). These results highlight the potential of targeting metabolic pathways as a strategic approach to modulate cardiac hypertrophy, proposing a novel therapeutic avenue for managing this condition.

## 4 DISCUSSION

Cardiac hypertrophy is associated with dysregulation of cardiomyocyte Ca^2+^, cell death, fibrosis, dysfunction of mitochondrial, metabolic changes, and fetal gene expression reactivation.^18^ Harmine, a naturally occurring alkaloid found in various plant species and a major component of the psychoactive brew ayahuasca, has demonstrated a broad range of biological and pharmacological activities, including DNA intercalation and inhibition of dual‐specificity tyrosine phosphorylation‐regulated kinase 1A (DYRK1A) and cyclin‐dependent kinases (CDKs).^22^, ^23^, ^24^ In our previous research, harmine emerged as a promising therapeutic candidate for mitigating cardiac hypertrophy by inhibiting NF‐κB phosphorylation.^1^ Selenomethionine, the primary organic selenium source, has been shown to inhibit cardiac autophagy and alleviate cardiac injury by modulating the AKT signalling pathway.^9^ Activation of the AKT signalling pathway enhances the transcriptional activity of NF‐κB, whereas inhibition of AKT disrupts NF‐κB activity.^10^ Additionally, selenium has been shown to exert epigenetic effects, modulating both DNA and histone structures to activate methylation‐silenced genes.^25^ Recent evidence highlights the critical role of post‐translational modifications (PTMs) in regulating myocardial hypertrophy, particularly through the addition (“writing”) or removal (“erasing”) of PTMs on histone proteins, which modulate key hypertrophic pathways.^26^ In the present study, we assessed the mRNA expression levels of ANP, BNP, and TNNT2 in cardiomyocytes to investigate the combined effects of SE and harmine in the treatment of cardiac hypertrophy. We observed that co‐treatment of SE and harmine at specific concentrations significantly attenuated cardiac hypertrophy in vitro and in vivo model. Through RNA sequencing analysis, we identified several keys signalling pathways affected by SE and harmine in cardiomyocytes. These include glycine, serine, and threonine metabolism; arachidonic acid metabolism; the p53 signalling pathway; the TNF signalling pathway; VEGF signalling pathway; viral protein interaction with cytokines and cytokine receptors; the JAK–STAT signalling pathway; the TGF‐β signalling pathway; and glutathione metabolism. It is worth noting that serine and glycine play vital roles in protein synthesis and cellular antioxidative regulation.^27^ The tumour protein p53 is associated with oxidative stress and regulates cellular proliferation in response to serine deprivation.^27^ Inhibiting p53 accumulation has been found to exacerbate hypertrophy under conditions of chronic pressure overload. These findings suggest that the anti‐angiogenic property of p53 may have a critical role in cardiac hypertrophy.^18^, ^28^ Interestingly, serine and threonine metabolism, along with the p53 signalling pathway, emerged as the key functions affected by SE and harmine in cardiomyocytes. Moreover, mRNA analysis revealed increased expression of BPGM, involved in glycine, serine, and threonine metabolism, while THBS1 and IGFBP3, involved in the p53 pathway, were found to be important in combating cardiac hypertrophy. These findings indicate that the combined treatment of SE and harmine may suppress cardiac hypertrophy by jointly modulating serine and threonine metabolism and the p53 signalling pathway. Arachidonic acid metabolism represents a critical signalling pathway associated with cardiac hypertrophy.^29^, ^30^ Our results support this suggestion, as we observed an increase in PTGS2 and GPX1, both involved in arachidonic acid metabolism, in cardiomyocytes following co‐treatment of SE and harmine. Tumour necrosis factor‐alpha (TNFα), a multifunctional cytokine, has been implicated as a mediator of cardiac pathology. In response to pressure overload‐induced cardiac hypertrophy, TNFα is upregulated in the adult heart.^31^, ^32^ TNF‐α mediates NF‐κB activity, thereby inducing cardiac hypertrophy. Conversely, specific inhibition of NF‐κB can downregulate TNF‐α‐induced inflammation in ventricular myocytes.^33^, ^34^ Interestingly, PTGS2, CCL2 and TNFRSF9 genes involved in the TNF signalling pathway, emerged as primary functions influenced by SE and harmine in cardiomyocytes. This suggests that the synergistic effect of SE and harmine against cardiac hypertrophy may be associated with the regulation of the TNF signalling pathway. VEGF is a pivotal angiogenic molecule that contributes to the maintenance of myocardial capillary density. Deletion of VEGF impairs myocardial angiogenesis and cardiac function, while inhibiting VEGF signalling promotes the transition to heart failure during pressure overload stimulation.^35^, ^36^ In our study, we observed changes in PTGS2 within the VEGF signalling pathway in cardiomyocytes, consistent with previous suggestions that PTGS2 is vital for the maintenance of healthy cardiac tissue.^37^ Cytokines, a group of modulatory proteins or glycoproteins, bind to their respective receptors to activate signal transduction pathways within cells and play pivotal roles in cardioprotection.^38^, ^39^, ^40^ In our study, the expression of CCL2 and CXCR4, both involved in viral protein interaction with cytokine and cytokine receptor interaction pathways, was increased. Meanwhile, CXCL11, a chemokine superfamily member in viral protein interaction with cytokine and cytokine receptor interaction pathways, was decreased under the same condition. The JAK–STAT intracellular signal transduction pathway is intricately linked to the activation of cytokine receptors and plays a central role in cardiac hypertrophy mediated by the AngII type 1 (AT1) receptor.^40^, ^41^ The RNA‐seq analysis and mRNA evaluation results further corroborated the significant involvement of the JAK–STAT signalling pathway in cardiac hypertrophy. TGF‐β, a pivotal member of the mitogen‐activated protein kinase (MAPK) family, can activate the extracellular signal‐regulated kinase (ERK) pathway, c‐Jun N‐terminal kinase (JNK) pathway, and p38 pathway within the MAPK pathway to regulate the development of myocardial fibrosis.^42^, ^43^, ^44^, ^45^, ^46^ Notably, the expression of THBS1, involved in the TGF‐β pathway, was up‐regulated following co‐treatment of SE and harmine in cardiomyocytes. These findings seemingly contradict a previous study that revealed the induction of cardiac hypertrophy by the activation of TGF‐β activated kinase 1.^47^ Glutathione, a precursor of the largest intracellular thiol pool responsible for combating oxidative stress, has been shown to reverse cardiac hypertrophy.^48^ Notably, GPX1, an important player in glutathione metabolism, exhibited increased expression following co‐treatment of SE and harmine, suggesting that inducing glutathione metabolism may serve as a potent strategy for cardioprotection.

A hypertrophic heart undergoes significant metabolic reprogramming alongside structural changes, characterized by a transition from fatty acid oxidation to glycolysis.^49^, ^50^ Our metabolic analysis revealed that glycolysis/gluconeogenesis, phosphonate and phosphinate metabolism, thermogenesis, and the calcium signalling pathway were enriched as primary functions affected by SE and harmine in cardiomyocytes. Glycolysis metabolism involves the conversion of glucose into pyruvate, generating small amounts of ATP and NADH. Phosphoenolpyruvic acid, derived from the enol of pyruvate and phosphate, plays a role in both glycolysis and gluconeogenesis. Phosphoenolpyruvate carboxykinase (PCK) is recognized as a critical enzyme in gluconeogenesis, as it is responsible for the production of phosphoenolpyruvic acid by phosphorylating oxaloacetic acid. Dysregulation of PCK is prominently observed in the development of cardiac hypertrophy.^50^, ^51^ In our study, we observed a decrease in phosphoenolpyruvic acid, involved in glycolysis/gluconeogenesis and phosphonate/phosphinate metabolism, following co‐treatment of SE and harmine. These findings indicate that the combined treatment of SE and harmine may suppress AngII‐induced cardiac hypertrophy through modulating carbohydrate metabolism and amino acid metabolism. In healthy cells, glycolysis leads to the reduction of NAD^+^ to NADH, while an elevation in NAD^+^ levels has been associated with cardiac hypertrophy.^52^, ^53^ In our study, we observed an increase in NADH levels within the thermogenesis and calcium signalling pathways following co‐treatment of SE and harmine in cardiomyocytes. These findings suggest that the combined treatment may suppress cardiac hypertrophy by reducing glycolysis metabolism. Joint analysis further supports these findings, indicating that glycolysis metabolism is the main pathway through which cardiac hypertrophy is suppressed following co‐administration of SE and harmine. Several critical enzymes involved in governing glycolysis, including hexokinase, phosphofructokinase, and pyruvate kinase, have been proposed due to their role in catalysing irreversible reactions within the glycolysis pathway.^54^ Hexokinase acts as the first enzyme of glycolysis, regulating glucose transport. Phosphofructokinase (PFK) is the second regulatory enzyme, responsible for converting fructose 6‐phosphate to fructose 1,6‐bisphosphate and fructose 2,6‐bisphosphate (fructose 2,6‐BP). Pyruvate kinase, the final enzyme of glycolysis, governs the flux through this pathway.^49^ To further validate the important role of glycolysis metabolism against cardiac hypertrophy, a series of key targets within the glycolysis metabolism were evaluated. The results demonstrated that co‐treatment of SE and harmine significantly decreased the production of lactic acid, pyruvate, glucose 6‐phosphate, the NAD^+^/NADH ratio, and ATP levels in AngII‐induced cardiac hypertrophy. Additionally, the reduced activity of GAPDH was observed by co‐treatment with SE and harmine in cardiomyocytes. Notably, when cardiac hypertrophic cells underwent co‐treatment with 2‐DG, the protective effects of SE and harmine was not enhanced by 2‐DG. These observations suggest that the combined therapeutic intervention of SE and harmine can significantly suppress cardiac hypertrophy mainly through the modulation of glycolysis metabolism.

## 5 CONCLUSION

In summary, this study highlights the potential of co‐treating SE and harmine as a promising therapeutic strategy against cardiac hypertrophy. Mechanistically, the combination treatment of SE and harmine demonstrated the ability to reduce hypertrophy‐related targets and cellular size of cardiomyocytes principally by suppressing glycolytic metabolism. These results suggest that the synergistic effects of SE and harmine hold promise as an effective approach against cardiac hypertrophy.

## AUTHOR CONTRIBUTIONS

**Qi Chen:** Conceptualization (equal); investigation (equal); methodology (equal); validation (equal). **Wen‐Yan Wang:** Investigation (equal); validation (equal). **Qing‐Yang Xu:** Validation (equal). **Yan‐Fa Dai:** Conceptualization (equal); investigation (equal); validation (equal). **Xing‐Yu Zhu:** Validation (equal). **Zhao‐Yang Chen:** Conceptualization (equal); data curation (equal). **Ning Sun:** Conceptualization (equal). **Chung‐Hang Leung:** Conceptualization (equal); visualization (equal). **Fei Gao:** Conceptualization (equal); investigation (equal); visualization (equal). **Ke‐Jia Wu:** Conceptualization (equal); funding acquisition (equal); project administration (equal); writing – original draft (equal); writing – review and editing (equal).

## FUNDING INFORMATION

This research was funded by National Natural Science Foundation of China (No. 82200403 and 82470364, China); and Postgraduate Research & Practice Innovation of Jiangsu Province (KYCX24_2646); Fundamental Research Funds for the Central Universities (22520231069).

## CONFLICT OF INTEREST STATEMENT

The authors confirm that this article content has no conflict of interest.

## Supporting information


Data S1.

## Data Availability

The original contributions presented in the study are included in the article/Supplementary Material, further inquiries can be directed to the corresponding author. Raw reads of the transcriptome project have been deposited in NCBI's BioProject accession number PRJNA996079.
