# Supplementary material for: The enhancing effects of selenomethionine on harmine in attenuating pathological cardiac hypertrophy via glycolysis metabolism
Source: J Cell Mol Med. 2024 Oct 1;28(19):e70124. doi: 10.1111/jcmm.70124 (PMC11443162; doi:10.1111/jcmm.70124)
Supplement: Supplementary file 1 — Data S1. [file JCMM-28-e70124-s001.docx]

Supplementary Material

The Enhancing Effects of Selenomethionine on Harmine in Attenuating Pathological Cardiac Hypertrophy via Glycolysis Metabolism

Qi Chen^1,#^, Wen-Yan Wang^1,#^, Qing-Yang Xu^1^^,2^, Yan-Fa Dai^1^, Xing-Yu Zhu^1^, Ke Zhang^1^, Zhao-Yang Chen^3^, Ning Sun^1,2^, Chung-Hang Leung^4,5^, Fei Gao^6,^*, Ke-Jia Wu^1,^*

^1^ Wuxi School of Medicine, Jiangnan University, 1800 Lihu Avenue, Wuxi, Jiangsu, 214122, P. R. China.

^2^ Department of Physiology and Pathophysiology, State Key Laboratory of Medical Neurobiology, School of Basic Medical Sciences, Fudan University, 138 Yi Xue Yuan Road, Shanghai, Shanghai, 200032, P. R. China

^3^ Department of Cardiology, Heart Center of Fujian Province, Fujian Medical University Union Hospital, 29 Xin-Quan Road, Fuzhou, Fujian, 350001, P. R. China.

^4^ State Key Laboratory of Quality Research in Chinese Medicine, Institute of Chinese Medical Sciences, University of Macau, Macao, 999078, P. R. China

^5^ Department of Biomedical Sciences, Faculty of Health Sciences, University of Macau, Macao, 999078, P. R. China

^6^ Department of cardiology, Beijing An Zhen Hospital, Capital Medical University, Chaoyang District, Beijing 100029, P.R. China

^#^ These authors contributed equally to this work.

**Supplementary methods**

**1. Human embryonic stem cells Culture and Treatment**

Human embryonic stem cells (hESCs) were cultured using mTeSR medium (STEMCELL Technologies, Cat#85851 and #85852) on Matrigel-coated plates (Corning, Cat#354230) and dissociated with Accutase (Sigma, Cat#A6964). Briefly, when the cells reached 80%-90% confluence on day 0, hESCs were treated with RPMI-1640 (Gibco)/B-27 minus insulin medium (ThermoFisher Scientific, Cat#A1895601) containing 8 μM CHIR-99021 (Selleck, Cat#S1263) for 2 days. Subsequently, fresh RPMI-1640/B-27 minus insulin medium supplemented with CHIR-99021 was provided at day 2 for 24 hours. From day 3 to day 5, the cells were treated with 5 μM IWR-1 (Sigma, Cat#I0161). Starting from day 6, the cells were cultured in RPMI-1640/B-27 medium with insulin, and the medium was refreshed every 2 days. Cardiomyocytes at day >25 were used and dissociated into single cells using Collagenase 1 (Sigma, Cat#SCR103) and 0.05% EDTA/Trypsin (Sigma, Cat#59417C).

**2. Calcium transient imaging**

Single hESC-CMs were reseeded onto 20 mm glass-bottom dishes in DMEM supplemented with 10% FBS. After a 24 hours co-treatment, the cardiomyocytes were treated with 5 μM Cal-520AM in Tyrode's solution containing 10% FBS at 37°C for 15 min. Calcium signaling of the cardiomyocytes was then recorded at 37°C using the line-scan mode (10 ms) of a Zeiss LSM880 laser scanning confocal microscope (Carl Zeiss). The acquired data were analyzed using MATLAB software.

**Supplementary figures**


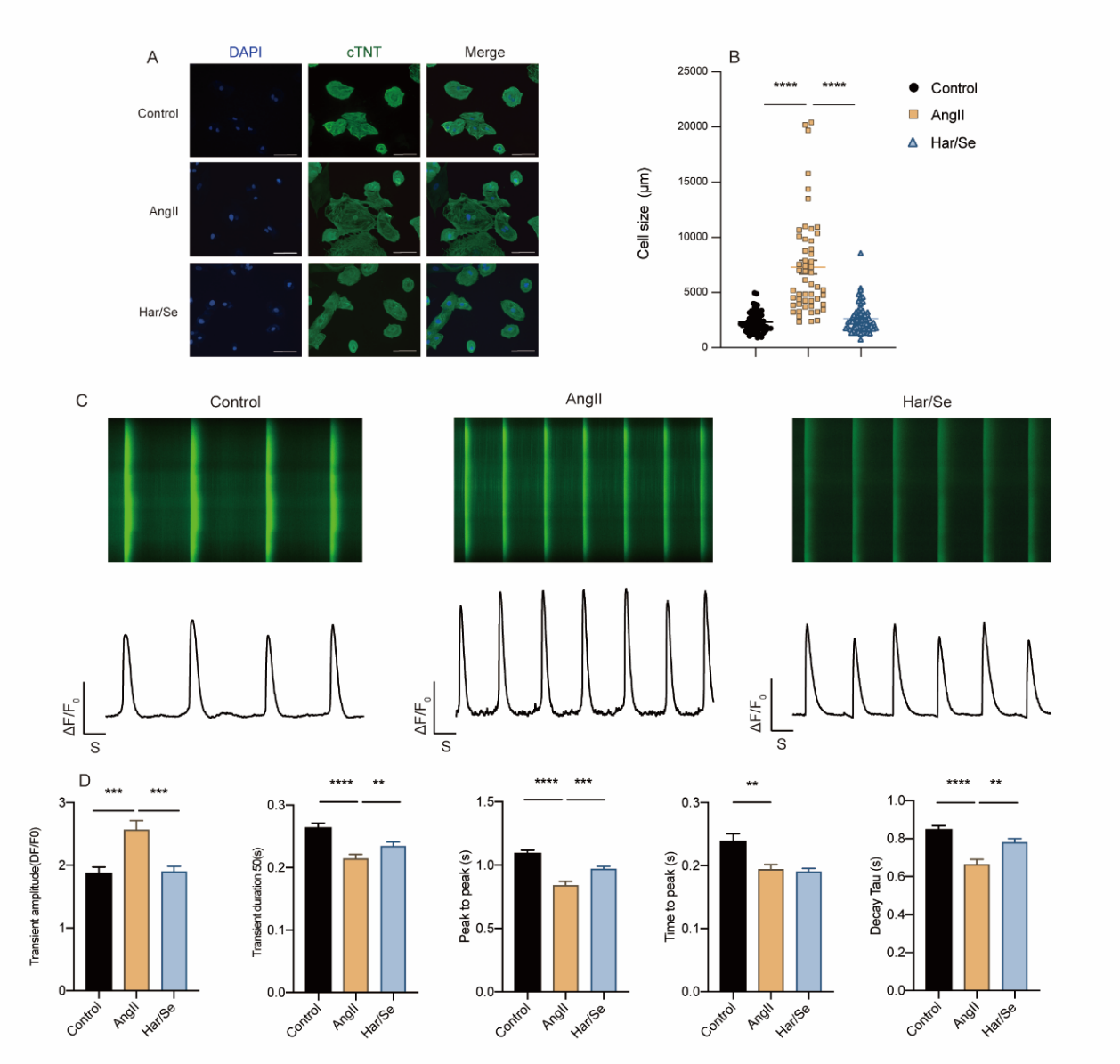


**Fig. S1** Co-treatment of SE and Harmine suppresses AngII-induced hypertrophic responses in hESC-CMs. (A) Representative immunofluorescence images of cTNT-stained for sarcomere marker (green) and DAPI (blue) for nuclei of hESC-CMs. Scale bar=20μm. (B) Quantification of cell area shown in A (n>50 cells per group). (C) Representative line scan images of calcium transients recorded in single cardiomyocyte. (D) Quantification of calcium handling parameters (transient amplitude (average ΔF/F0), transient duration 50, peak to peak time, time to peak, decay time) in single cardiomyocyte 24h after Ang I with or without SE and Harmine treatment (n>50 cells per group). Data are presented as mean±SEM. ***p*<0.01, ****p*<0.001, *****p*<0.0001 versus control by two-tailed Student’s t test. Har is an abbreviation of Harmine, Se is an abbreviation of selenomethionine.

**
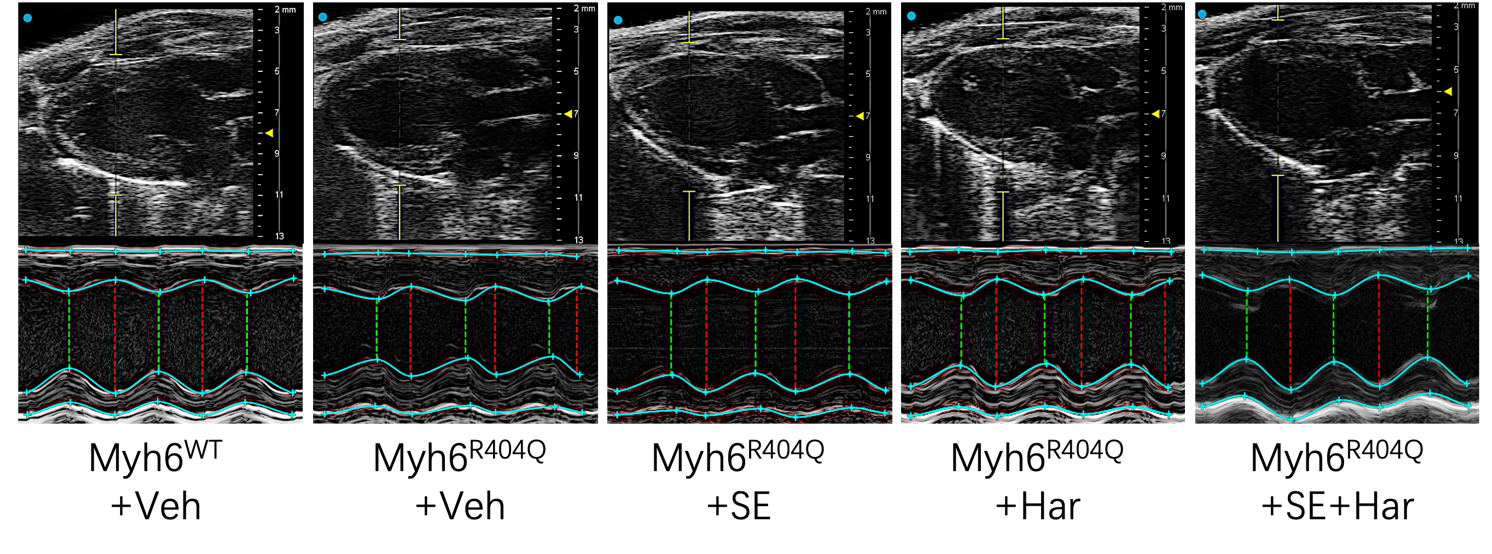
 Fig. S2** The representative images of echocardiography.

**Supplementary Table 1.** Primers used in RT-qPCR analysis for AC16 cell.

| Primer | Forward | Reverse |
| --- | --- | --- |
| 36B4 | AGCCACGCTGCTGAACAT | CAACATTGCGGACACCCT |
| PTGS2 | CGGTGAAACTCTGGCTAGACAG | GCAAACCGTAGATGCTCAGGGA |
| CCL2 | CAGCCAGATGCAATCAATGCC | TGGAATCCTGAACCCACTTCT |
| THBS1 | TGACAACAACGTGGTGAATGG | ACAGGAGATGCCGCAGATG |
| INHBA | CCTCCCAAAGGATGTACCCAA | CTCTATCTCCACATACCCGTTCT |
| IGFBP3 | ACCTGAGATGAGACAGGAGTC | GTAGAATCCTTTGCGGTCACAA |
| GPX1 | CAGTCGGTGTATGCCTTCTCG | GAGGGACGCCACATTCTCG |
| BPGM | TGCTTGGAATAAGGAGAACCGT | CCACAGTTCCGAGCTTCCTC |
| PDGFB | CTCGATCCGCTCCTTTGATGA | CGTTGGTGCGGTCTATGAG |
| G6PD | CGAGGCCGTCACCAAGAAC | GTAGTGGTCGATGCGGTAGA |
| HK1 | GCTCTCCGATGAAACTCTCATAG | GGACCTTACGAATGTTGGCAA |
| PFKM | GGTGCCCGTGTCTTCTTTGT | AAGCATCATCGAAACGCTCTC |
| PGK1 | TGGACGTTAAAGGGAAGCGG | GCTCATAAGGACTACCGACTTGG |
| ENO1 | AAAGCTGGTGCCGTTGAGAA | GGTTGTGGTAAACCTCTGCTC |
| PKM2 | ATGTCGAAGCCCCATAGTGAA | TGGGTGGTGAATCAATGTCCA |
| LDHA | ATGGCAACTCTAAAGGATCAGC | CCAACCCCAACAACTGTAATCT |
| LDHB | TGGTATGGCGTGTGCTATCAG | TTGGCGGTCACAGAATAATCTTT |
| CXCR4 | ATATACACTTCAGATAACTACACCGAG | TCAGTTTCTTCTGGTAACCCATGACCA |
| CXCL11 | GACGCTGTCTTTGCATAGGC | GGATTTAGGCATCGTTGTCCTTT |
| TNFRSF9 | CTCACGCTCCGTTTCTCTGT | GAAATCGGCAGCTACAGCCA |
